# Numerical simulation of closed plastic impeller molding process and its parameter optimization

**DOI:** 10.1038/s41598-022-22260-7

**Published:** 2022-10-15

**Authors:** Mingyue Fang, Zhaozhe Zhu, Zhendong Zhang

**Affiliations:** 1 Anhui Institute of Information Technology, Wuhu, 241100 Anhui China; 2 College of Mechanical Engineering, Anhui Polytechnic University, Wuhu, 241000 Anhui China

**Keywords:** Mechanical engineering, Applied mathematics

## Abstract

Aiming at the warping deformation and volume shrinkage of the closed plastic impeller, the relevant compression molding process parameters were optimized.Based on the theoretical equation of the pressing pressure for general sheet plastics, the theoretical derivation of the pressing pressure suitable for cylindrical cavities was carried out, and the theoretical derivation of the pressing pressure suitable for closed plastic impeller molding was derived, and the pressing pressure was calculated to be 15 MPa. The warpage deformation and shrinkage rates under different combinations of process parameters were obtained, and the influence of process parameters on warpage deformation and shrinkage rates, as well as the two combinations of process parameters that satisfy the best warpage deformation and shrinkage rates, respectively, were obtained by extreme difference analysis. A GA-BP neural network model was established for the prediction of the process parameters of closed plastic impeller compression molding, and the prediction curves were fitted into a function to obtain a set of process parameter combinations with optimal warpage and shrinkage at the same time by using the multi-objective optimization function of NSGA-II algorithm.

## Introduction

The closed impeller is an important working element of the centrifugal pump and the core flow-through component^1^. Its manufacturing technology and quality directly affect the hydraulic performance, cavitation performance, and operation stability of the pump. As one of the main molding processes of the closed plastic impeller, molding has the advantages of simpler mold structure, better dimensional accuracy, and surface smoothness compared with injection molding^2^. Manufacturing defects such as volume shrinkage and warpage will occur in the molding process of the parts, and the process parameters are one of the important factors affecting the volume shrinkage and warpage of the parts^3–5^. In order to reduce the volume shrinkage and warpage of the parts, the selection of process parameters has been optimized by constant mold trial and mold repair, but this method has not met the requirements of modern design^6^. The intelligent algorithm is a key link and quality assurance of intelligent industrial manufacturing, and the advantages of the intelligent algorithm in optimizing parameters are applied in many fields. The process parameters can be optimized through the combination of an intelligent algorithm and CAE software so as to reduce the warping deformation and volume shrinkage of plastic parts^7^.

Xie Huaiqin et al.^8^ performed theoretical analysis and calculation based on the theory of curing kinetics and heat transfer, used DSC experiments to obtain the raw data of curing kinetics, and fused both finite difference and finite unit methods. The mathematical model of the relationship between the curing degree values and the temperature field was designed, and the Euler iteration method was proposed to decouple the mathematical model. The relationship between the curing degree and the unsteady temperature field is determined by numerical simulation, which provides sufficient theoretical guidance for the selection of the compression molding process.

Jianhan Zhang et al.^9^ investigated textile composites based on the stereoscopic compression molding properties of three-dimensional surfaces, and numerically simulated the hemispherical molding of the material by the NURBS method. For the shortcomings of the traditional NURBS method, the use of NURBS interpolation curves was proposed to analyze the material forming. The spatial coordinate positions of the warp and weft yarn interweaving points in the fabric under three-dimensional conditions are obtained, which provide a theoretical basis for the prediction of molding defects affecting the product quality.

Kamran^10^ used hot compression molding to optimize PLA preparation and PLA samples to obtain PLA with the highest tensile strength, flexural strength, and hardness values. The role of different processing variables such as temperature, pressure, compression time, and holding and cooling time on PLA molding was investigated. These variables were investigated to optimize PLA properties and to produce composite samples using the optimized processing parameters.

Some scholars only numerically simulate the molding process alone, and there are few in-depth studies on the molding process parameters of plastic materials; some scholars only experimentally study the effects of different molding processes on the molding quality and cannot comprehensively and completely numerically simulate the molding process and optimize the molding process parameters. In this paper, theoretical calculations and the design of a closed plastic impeller are carried out to obtain a closed-type plastic impeller with better molding quality. The orthogonal experiments on the process parameters and the numerical simulation of the heating process and compression molding process using the ANSYS function are carried out to obtain a set of process parameters with better warpage deformation and shrinkage.

## Centrifugal pump closed plastic impeller molding process parameters and theoretical derivation of compression molding force

### Centrifugal pump closed plastic impeller molding

The main parameters of the plastic centrifugal pump are shown in Table 1.

**Table 1:** Main parameters of plastic centrifugal pumps.

Parameters	Flow rate Q(m^3^/h)	Lift H(m)	Rotational speed n(r/min)
Numerical values	30	10	1450

According to the main parameters of the plastic centrifugal pump, the structural parameters of the closed plastic impeller are calculated and designed. The structural parameter design of the closed plastic impeller is the most fundamental and important part of the process of its molding die technology research and parameter optimization. The structural parameters are calculated by the formula, and the calculated parameters are shown in Table 2.

**Table 2:** Design parameters for closed plastic impellers.

Performance parameters		Geometric dimensions	
Flow rate Q(m^3^/h)	30	Impeller inlet diameter(mm)	80
Lift H(m)	10	Impeller outlet diameter(mm)	200
Rotational speed n(r/min)	1450	Blade inlet width(mm)	26
Specific speed n_s_	85.92	Blade outlet width(mm)	30
Volute width(mm)	75	Blade inlet installation angle(°)	15
Diameter of spiral case base circle(mm)	216	Blade outlet installation angle(°)	40
Number of blades	6	Wrap angle(°)	94

**Figure 1:**
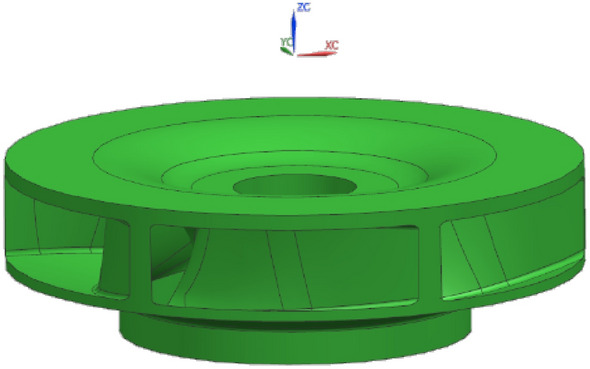
3D model of a closed plastic impeller.

The 3D model of the closed plastic impeller is obtained by 3D modeling with UG, as shown in Fig. 1.

Closed-type plastic impeller molding is done through the mechanical pressure generated by a hydraulic press to make the concave and convex mold close the mold, thus pressing the plastic material into the required shape. The closed-type plastic impeller molding process is to heat the mold to a certain temperature and then add the preheated plastic material through the charging chamber. The plastic is heated to a given heating temperature, pressed, and cooled after a certain time of holding pressure, and finally, the product is obtained after demolding^11–17^.

### Selection of molding process parameters for closed plastic impeller

#### Heating temperature

The heating plate is used to heat the convex die of the closed plastic impeller pressing mold, and the heating ring is used to heat the concave die of the closed plastic impeller pressing mold so that the closed plastic impeller mold and the plastic material can reach the necessary pressing and molding temperature^18^. The proper temperature not only heats the plastic material faster with quality assurance, but also reduces the pressing time of the closed plastic impeller, making the production process compact and efficient.

The main component of the plastic raw material used in the closed plastic impeller is UTR9000, whose melting point is generally about 170 °C and the decomposition temperature is about 260 °C. The closed plastic impeller molding mainly relies on the UTR9000 material becoming molten. The heating temperature is based on the UTR9000 molding temperature and ensures that the temperature is not too high to cause the decomposition of UTR9000.

The heating process conforms to Eq. (1) Fourier law:

1 $$ q^{*} = - Knn\frac{\partial T}{{\partial n}} $$

where, ${q}^{*}$—Heat flux density, $\mathrm{W}/{\mathrm{m}}^{2}$; ${k}_{nn}$—thermal conductivity, W/m °C, Along the $\frac{\partial T}{\partial n}$ temperature gradient, the minus sign means that heat is transferred from places with high temperature to places with low temperature.

According to the transient thermal analysis $\mathrm{q}=\frac{\mathrm{du}}{\mathrm{dt}}$ (indicating that the rate of heat conduction inflow or outflow $\mathrm{q}$ is equal to the change of internal energy of the system), during the heating process of closed plastic impeller compression molding, the whole compression mold system is divided into micro elements, and the governing differential equation of heat conduction of closed plastic impeller compression mold can be obtained by Eq. (2):

2 $$ \frac{\partial }{\partial x}\left( {k_{xx} \frac{\partial T}{{\partial x}}} \right) + \frac{\partial }{\partial y}\left( {k_{yy} \frac{\partial T}{{\partial y}}} \right) + \frac{\partial }{\partial z}\left( {k_{zz} \frac{\partial T}{{\partial z}}} \right) + \overline{q} = \rho c\frac{dT}{{dt}} $$

where, $\overline{q }$—Heat generation per unit volume, $\mathrm{J}/{\mathrm{m}}^{2}$; $\uprho$—Material density,$\mathrm{kg}/{\mathrm{m}}^{3}$; $\mathrm{c}$—Specific heat capacity of material, ${\text{J}}/{\text{kg}},$°C; $\frac{dT}{dt}$—Heat changes with time.

$\frac{dT}{dt}$ can be obtained from Eq. (3):

3 $$ \frac{dT}{{dt}} = \frac{\partial T}{{\partial t}} + V_{x} \frac{\partial T}{{\partial x}} + V_{y} \frac{\partial T}{{\partial y}} + V_{z} \frac{\partial T}{{\partial z}} $$

where, ${V}_{x},{V}_{y},{V}_{z}$—Media conductivity; $\frac{\partial T}{\partial x}, \frac{\partial T}{\partial y}, \frac{\partial T}{\partial z}$—Temperature gradients along directions $\mathrm{x},\mathrm{y},\mathrm{z}$ respectively.

Equation (4) can be obtained by integrating both sides of Eq. (3):

4 $$ \begin{gathered} \int_{vol} {\left[ {\rho c\delta T\left[ {\frac{\partial T}{{\partial t}} + \left\{ v \right\}^{T} \left\{ L \right\}^{T} } \right] + \left\{ L \right\}^{T} \delta T\left( {\left[ D \right]\left\{ L \right\}^{T} } \right)} \right]} d\left( {vol} \right) \hfill \\ \quad \quad \quad \quad \quad = \int_{{S_{2} }} {\delta Tq^{*} d\left( {S_{2} } \right) + } \int_{{S_{3} }} {\delta Th_{f} \left( {T_{B} - T} \right)d\left( {S_{3} } \right) + \int_{vol} {\delta T\overline{q}d\left( {vol} \right)} } \hfill \\ \end{gathered} $$

where, $\overline{q }$—Heat generation per unit volume, $\mathrm{J}/{\mathrm{m}}^{3}$; ${h}_{f}$—Convective heat transfer coefficient; ${T}_{B}$—Temperature of fluid, ℃; $\mathrm{\delta T}$—Virtual variable of temperature, ℃; ${S}_{2}$—Area of applied heat flux, ${m}^{2}$; ${S}_{3}$—Convection applied area, ${m}^{2}$.

Equation (4) is the mathematical model of the heating process of a closed plastic impeller at a certain heating temperature. The heating process of plastic raw materials at a certain heating temperature is simulated by the transient thermal analysis module in a workbench^19^.

#### Heating time

The heating time is the time when the closed plastic impeller is heated to the time when it can be molded. The closed plastic impeller molding mainly heats the UTR9000 material into a molten state, and the heating time is based on the UTR9000 molding temperature. Through the numerical simulation and analysis of the transient thermal analysis module in the workbench, the change of the temperature of the plastic raw material in the closed plastic impeller mold with the heating time can be obtained. The appropriate heating time can ensure that the temperature of the plastic raw material in the mold reaches a more appropriate molding temperature.

#### Pressing pressure

The pressing pressure is the force exerted on the plastic raw material after the upper and lower mold of the closed plastic impeller is closed^20^. The heating temperature of compression molding can be reduced by appropriately increasing the compression molding pressure, so that the product density is appropriate, the dimensional accuracy and the surface contour are clear, but the compression molding pressure should not be too large, otherwise the service life of the closed plastic impeller mold will be reduced and the residual stress in the closed plastic impeller molding parts will be increased.

#### Pressing time

In the process of thermoplastic pressing, it is necessary to apply pressure to the mold and keep it for a period of time to ensure that the closed plastic impeller is fully formed^21^. Proper pressing time can ultimately produce high-quality products, which is of great help in reducing the warpage deformation, and shrinkage of the closed plastic impellers and improving surface gloss, physical and mechanical properties.

### Theoretical derivation of compression molding force of closed plastic impeller

**Figure 2:**
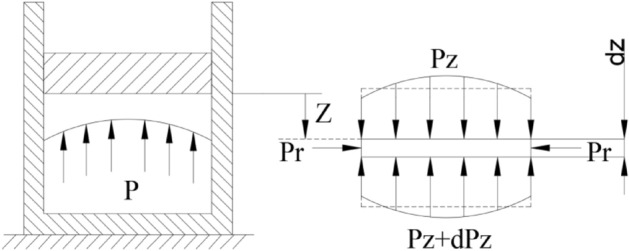
Material pressure distribution in mold.

In the case of neglecting the blade parting, the cavity of the mold can be approximated as a cylinder. The inner diameter of the cavity is R, and the mold direction is Z. The uniform pressure in this direction is set to P_z_, the radial uniform pressure is set to P_r_, and a plastic material microelement d_z_ in the closed plastic impeller cavity is taken for analysis. The pressure distribution has a pressure gradient, as in Fig. 2.

**Figure 3:**
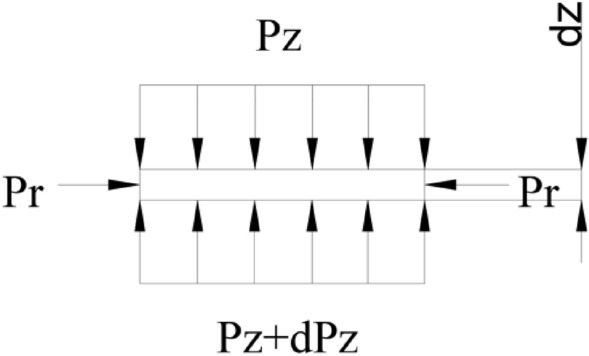
Approximate average pressure of material in mold.

Simplify the theoretical model and approximate the pressure P_Z_ to the average pressure on the section, as shown in Fig. 3.

According to the force balance Eq. (5) of Z-direction pressure distribution:

5 $$ P_{Z} A - \left( {P_{Z} + dP_{Z} } \right)A = \tau \pi Rd_{Z} $$

where, ${\mathrm{P}}_{\mathrm{Z}}$—Uniform pressure above micro ${\mathrm{d}}_{\mathrm{Z}}$, $\mathrm{MPa}$; $\left({\mathrm{P}}_{\mathrm{Z}}+{\mathrm{dP}}_{\mathrm{Z}}\right)$—Uniform pressure under micro ${\mathrm{d}}_{\mathrm{Z}}$, $\mathrm{MPa}$; $\mathrm{A}$—Bottom area of closed plastic impeller cavity cylinder, ${\mathrm{mm}}^{2}$; $\uptau$—Shear force of the cavity of the closed plastic impeller with plastic raw materials in Z direction, N; $\mathrm{R}$—Inner diameter of closed plastic impeller cavity, $\mathrm{mm}$; Z—Distance between micro element ${\mathrm{d}}_{\mathrm{Z}}$ and the bottom of closed plastic impeller punch, $\mathrm{mm}$.

By simplifying the above equation, Eq. (6) can be obtained:

6 $$ \frac{{dp_{z} }}{dZ} = - \frac{4\tau }{R} $$

According to Eq. (7) Coulomb friction law:

7 $$ \tau = \eta P_{r} $$

where, $\upeta$—The contact friction coefficient between the inner wall of the cavity of the closed plastic impeller and the plastic raw material; ${\mathrm{P}}_{\mathrm{r}}$—The uniformly distributed pressure of plastic raw materials in the radial direction, $\mathrm{MPa}$.

The value of ${\mathrm{P}}_{\mathrm{r}}$ is obtained from Eq. (8):

8 $$ P_{r} = aP_{Z} $$

Substitute Eq. (8) into Eq. (7) to obtain Eq. (9):

9 $$ \tau = a\eta P_{Z} $$

where, $a$—Relationship function between radial force and axial force.

Substitute Eq. (9) into Eq. (6) to obtain Eq. (10):

10 $$ \frac{{dP_{Z} }}{{P_{Z} }} = - \frac{4a\eta dZ}{R} $$

Let $a$ and $\eta$ be constants and integrate to obtain Eq. (11):

11 $$ \int_{{P_{0} }}^{{P_{Z} }} {\frac{{dP_{z} }}{{P_{z} }} = } - \int_{0}^{z} {\frac{4a\eta dZ}{R}} $$

By substituting boundary conditions $\mathrm{Z}=0$ and ${P}_{Z}={P}_{0}$ into Eq. (11), Eq. (12) can be obtained:

12 $$ \ln P_{Z} |\begin{array}{*{20}c} {P_{Z} } \\ {P_{0} } \\ \end{array} = - \frac{4a\eta Z}{R} $$

Simplified Eq. (12), available Eq. (13):

13 $$ P_{Z} = P_{0} e^{{ - \frac{4a\eta Z}{R}}} $$

The value of ${Z}_{0}$ is shown in Eq. (14):

14 $$ Z_{0} = \frac{R}{4a\eta } $$

Substituting Eq. (14) into Eq. (13), the equation for calculating the pressure of closed plastic impeller molding can be obtained, as shown in Eq. (15):

15 $$ P_{z} = P_{0} e^{{ - \frac{Z}{{Z_{0} }}}} $$

Equation (15) shows the pressure distribution of the plastic material in the closed plastic impeller cavity in the Z-direction, and the distribution shows obvious exponential characteristics. If the heating temperature of the plastic material in the closed plastic impeller is low, higher pressing pressure is needed, and if the heating temperature is high, the pressing pressure needs to be reduced appropriately.

In this paper, the inner diameter of the closed plastic impeller cavity is R = 100 mm, the relationship function between the radial pressure and axial pressure a, and the product of the contact friction coefficient n between the inner wall of the closed plastic impeller cavity and the plastic material a_η_ = 0.1, the distance between the plastic material and the bottom of the convex mold of the closed plastic impeller Z = 150 mm, P_0_ = 27 MPa, substituting into Eq. (15), we can get the closed plastic impeller compression molding The pressure is 14.82 MPa, rounded to 15 MPa.

### Determination of orthogonal test factors and levels of compression molding

Take a group of process parameters of the closed plastic impeller molding process as a group of factors of the orthogonal test, and select the experimental range of process parameters, as shown in Table 3.

**Table 3:** Factor level table of orthogonal test.

Level	Factors			
	A	B	C	D
	Heating temperature(°C)	Heating time(s)	Pressing pressure(MPa)	Pressing time(s)
1	170	500	15	60
2	180	600	20	70
3	190	700	25	80
4	200	800	30	90

Based on the principle of orthogonal test, orthogonal test table ${L}_{16}\left({4}^{5}\right)$ is established, as shown in Table 4.

**Table 4:** Orthogonal experiment arrangement.

Test no	A	B	C	D
1	170	500	15	60
2	170	600	20	70
3	170	700	25	80
4	170	800	30	90
5	180	500	20	80
6	180	600	15	90
7	180	700	30	60
8	180	800	25	70
9	190	500	25	90
10	190	600	30	80
11	190	700	15	70
12	190	800	20	60
13	200	500	30	70
14	200	600	25	60
15	200	700	20	90
16	200	800	15	80

## Numerical simulation of closed plastic impeller compression molding

Sixteen sets of process parameters from the orthogonal tests were analyzed by numerical simulation to obtain the simulation results corresponding to each set of process values, which are the warpage deformation of the closed plastic impeller obtained under each set of experimental data.

### Model import and material setting

The material used for the closed plastic impeller in this paper is URT9000 resin, which is a modified ABS-like resin with excellent durability, excellent chemical stability, and excellent mechanical properties. It is widely used in machinery, automobile, electrical appliances, and other fields^22^. Table 5 shows the performance parameters of URT9000 ABS resin.

**Table 5:** Performance parameters of resin materials (mechanical properties of cured materials).

Test items	Density (kg/m^3^)	Hardness (ShoreD)	Flexural modulus (MPa)	Flexural Strength (MPa)	Tensile modulus (MPa)	Tensile strength (MPa)	Thermal deformation temperature (°C)	Coefficient of thermal expansion (°C)
Value	1160	83	2692–2775	69–74	2189–2395	27–31	52	97*E-6

**Figure 4:**
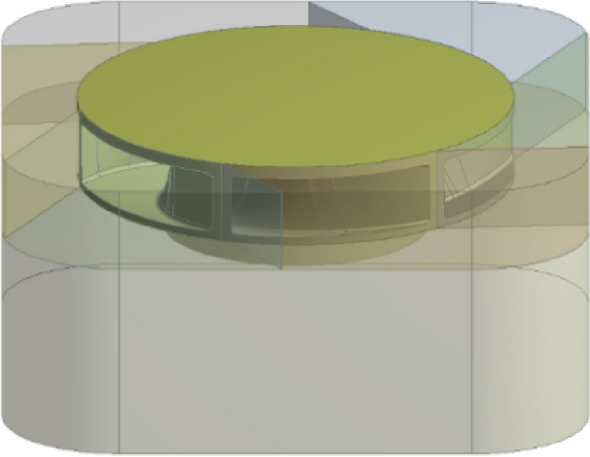
Model import.

**Figure 5:**
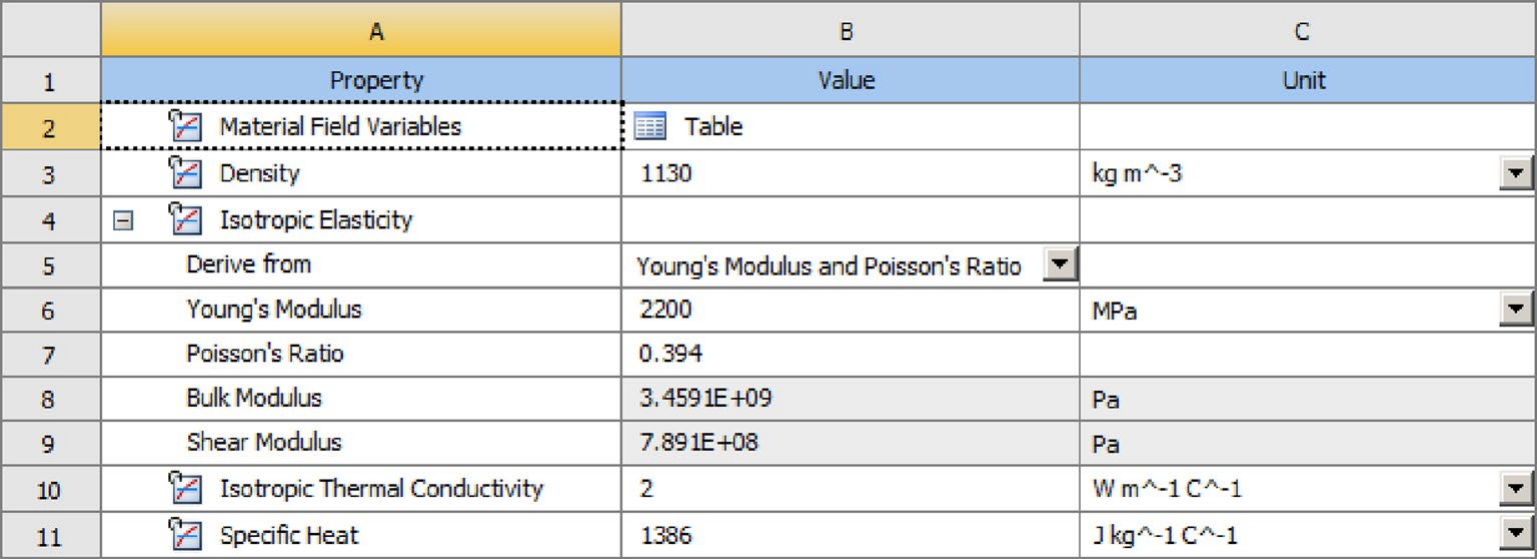
UTR9000 material parameters.

With the help of the Ansys Workbench platform, the closed plastic impeller was simulated numerically with thermal coupling. The model was first imported into the model module Geometry, as shown in Fig. 4. Then each of the imported bodies was numbered to facilitate the selection of parameters and meshing for the subsequent simulation. The material parameters are set in the material module Engineering Date, and the UTR9000 material parameters are shown in Fig. 5.

### Heating process simulation

#### Grid division and grid quality inspection

**Figure 6:**
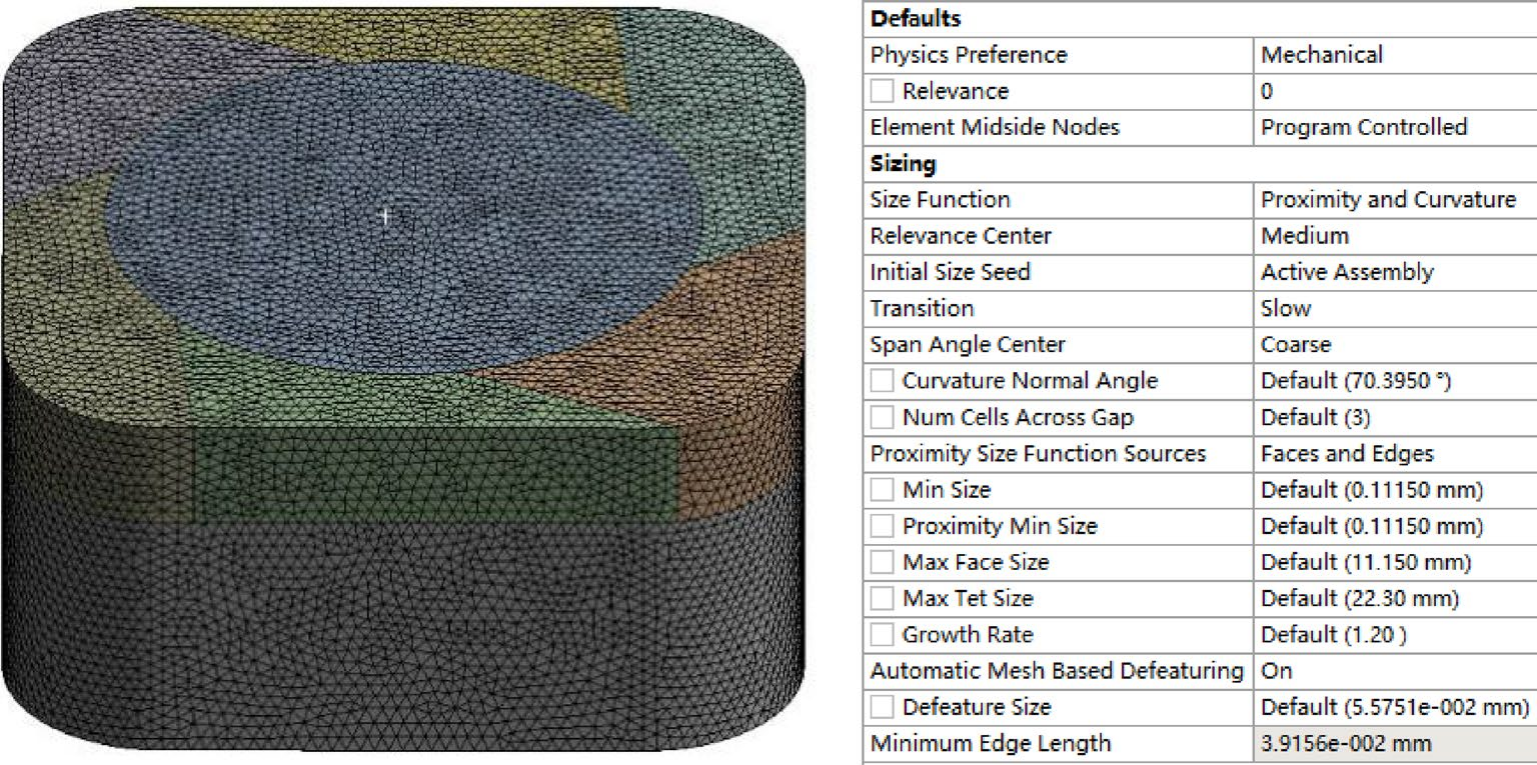
Mesh division and relevant details of closed plastic impeller and mold.

As there are many curved surfaces on the impeller and die, and the structure is relatively complex, this paper adopts unstructured mesh division^23^. Under the mesh project of the project, first insert the patch forming method, select tetrahedrons for the method in details of "patch forming method," then insert body sizing under the mesh project, and enter 5 mm for element size; In details of "mesh," click size function in sizing, select proximity and curvature, select medium in correlation center, and select slow in transition. Other settings are shown in Fig. 6.

The average quality of the results in the < Element Quality > module reached 0.84, which is within the range; the average value of the results in the < Jacobian Ratio > module reached 1.0029, which is within the range; the average value of the results in the < Skewness > module reached 0.21808, which is within the range. The analysis shows that the mesh quality meets the requirements, so the heating process of the plastic material in the mold can be simulated numerically.

#### Analysis settings

**Figure 7:**
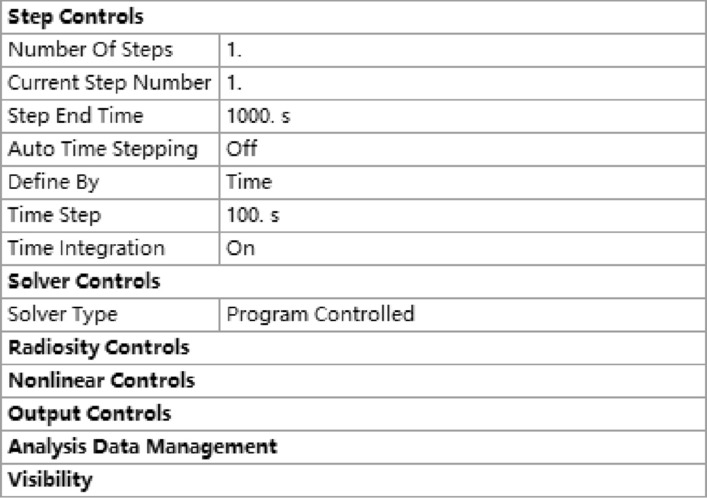
Analysis settings.

**Figure 8:**
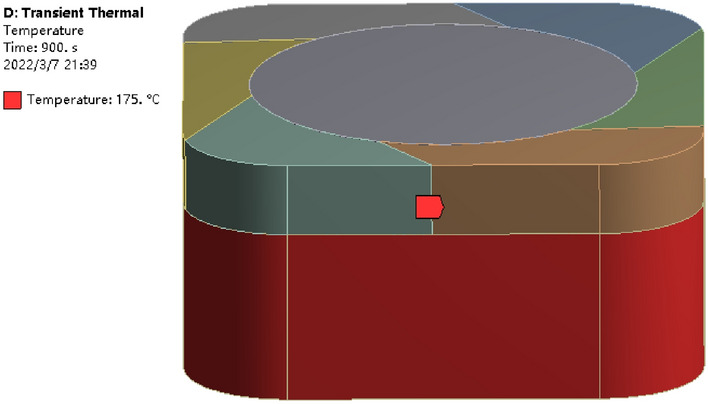
Temperature setting.

The mold material was selected as 45 gauge steel, and the plastic material was selected as UTR9000. In the heating stage, the heat of UTR9000 mainly comes from the high-temperature nitrogen in the molding furnace, thus defining the thermal boundary conditions. Set the < initial temperature value > to 80 °C. Set the step end time > to 1000 s and the < time step > to 100 s. The other settings remain unchanged, as shown in Fig. 7. The set temperature is 80 °C at 0 s, 170 °C at 1 s, and 170 °C at 1000 s; that is, the initial temperature of the mold and material is 80 °C, and the molding temperature in the furnace is 170 °C. Due to the high thermal conductivity and strong heat absorption capacity of the metal mold, it can rise to 170 °C in a relatively short time. During the heating process, put the impeller and the mold into the heating workstation for heating. The electric heating plate directly heats the upper and lower molds, so the heating boundary is the whole mold surface. After the temperature is set, it is shown in Fig. 8.

#### Solution setting and simulation calculation result analysis

**Figure 9:**
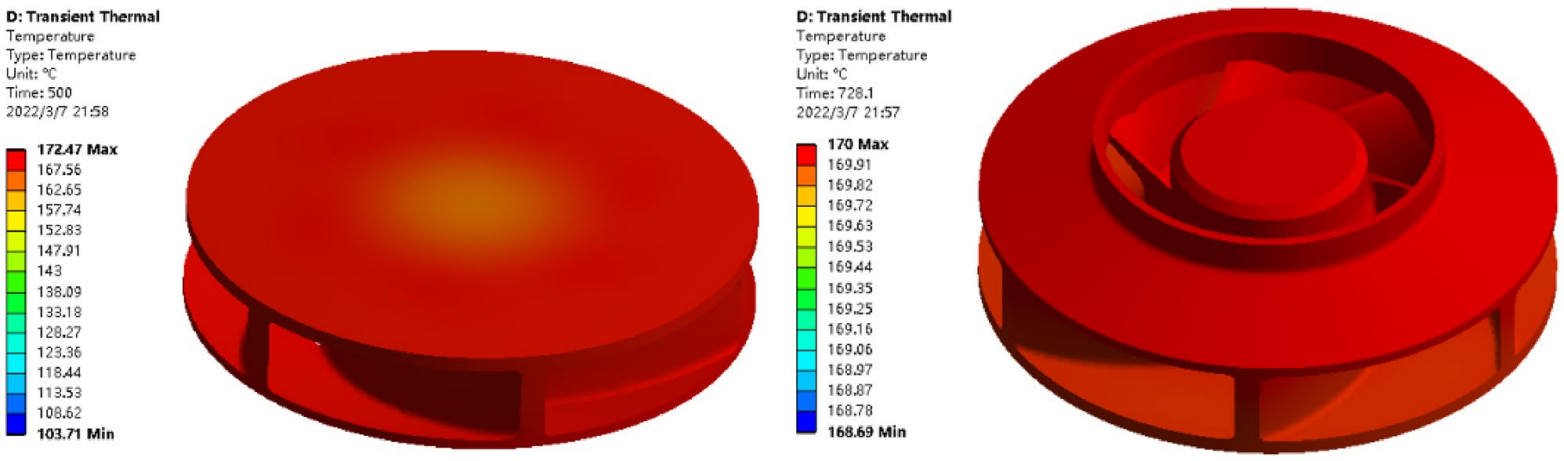
Simulation calculation results.

The transient thermal analysis was performed to obtain the molding temperature that the plastic raw material reaches by heating for a certain time at a certain heating temperature^24^. The specific value of the heating time is obtained by simulation and the temperature field data of the thermal coupling are obtained, and the simulation results are shown in Fig. 9. Through the simulation analysis, the plastic raw material shows a gradual change in temperature during the heating process, and the set molding temperature of 170 °C can be reached. The transient thermal analysis can effectively avoid the problem of edge rupture caused by molding the plastic raw materials before they all reach the pressing temperature, and there is no need to determine the time parameters of the heating stage.

### Simulation of compression molding process

#### Analysis settings

The pressing time was set to 60 s, and the pressing pressure was set to 15 MPa. During the molding process, as the inner surface of the product is close to the surface of the mold, the normal displacement of the entire inner surface of the product is considered to be constrained, and the molding pressure is applied to the outer surface of the convex mold. The transient thermal analysis simulation was carried out to obtain the thermodynamic coupled temperature field data loading.

The closed plastic impeller model is solved to obtain the numerical simulation results of the closed plastic impeller compression molding process under thermodynamic coupling conditions.

#### Solution setting and simulation calculation result analysis

**Figure 10:**
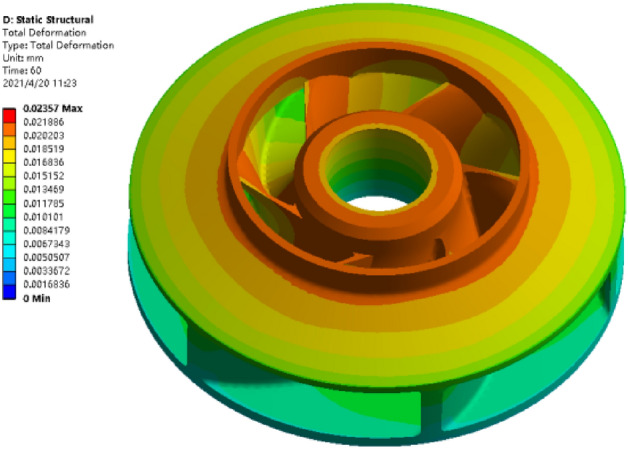
Simulation calculation results.

The purpose of the structural analysis is to obtain the two evaluation indexes of warpage deformation and shrinkage of the closed plastic impeller under a certain set of process parameters, and the simulation results of the structural analysis are shown in Fig. 10.The simulation analysis shows that the overall deformation distribution and the maximum deformation of the closed plastic impeller occur at the inlet of the front cover of the closed plastic impeller and at the blade connected to the inlet.

## Closed plastic impeller compression molding orthogonal test

The numerical simulation of the whole molding process of the closed-type plastic impeller was carried out with a certain set of process parameters as an example. According to the simulation results obtained, the warpage deformation and shrinkage rate are selected as the evaluation indexes of closed type plastic impeller molding, and the numerical simulation of the molding process is carried out for 16 sets of process parameter combinations of plastic raw materials of closed type plastic impeller, respectively.

### Measurement of each evaluation index of closed plastic impeller

#### Warpage deformation of closed plastic impeller

The deformation direction is consistent with the direction of the warpage deformation gradient, and the warpage deformation is equal to the maximum value of the gradient, as shown in Eq. (16), i.e. the warpage deformation parameters obtained by ANSYS simulation.

$$ l_{\max } = l_{1} - l_{2} $$

16 $$ \Delta l = l_{\max } $$

where, $l_{1}$—The coordinate value of maximum value point after molding, $l_{2}$—The coordinate value of maximum value point in an ideal state.

#### Shrinkage of closed plastic impeller

The linear distance between the mold corresponding to the point position of the maximum value and the mold corresponding to the other side with the same warping deformation gradient direction is a. The Shrinkage equation of the closed plastic impeller is shown in Eq. (17).

17 $$ \eta = \frac{\Delta l}{x} $$

where, $x$—the wall thickness of the workpiece corresponding to the point of the maximum value, $\eta$—Shrinkage.

The warpage deformation and shrinkage of the closed plastic impeller for each combination of process parameters can be obtained by Eq. (16) and Eq. (17), as shown in Table 6.

**Table 6:** Closed plastic impeller compression molding orthogonal test results.

Number	Heating temperature (°C)	Heating time (s)	Pressing pressure (MPa)	Pressing time (s)	Warpage deformation (mm)	Shrinkage (%)
1	170	500	15	60	0.0235	0.671
2	170	600	20	70	0.0314	0.785
3	170	700	25	80	0.0393	0.561
4	170	800	30	90	0.0471	0.693
5	180	500	20	80	0.0330	0.471
6	180	600	15	90	0.0251	0.418
7	180	700	30	60	0.0487	0.696
8	180	800	25	70	0.0409	0.584
9	190	500	25	90	0.0385	0.550
10	190	600	30	80	0.0464	0.663
11	190	700	15	70	0.0228	0.570
12	190	800	20	60	0.0306	0.437
13	200	500	30	70	0.0481	0.668
14	200	600	25	60	0.0402	0.731
15	200	700	20	90	0.0322	0.685
16	200	800	15	80	0.0244	0.610

###  Analysis of orthogonal experimental results of closed plastic impeller

In order to obtain the degree of influence of the process parameter combination on the two evaluation indexes and the process parameter combination under the best situation of both evaluation indexes, polar difference analysis is required for each result of the closed plastic impeller compression molding orthogonal experiment. The larger the extreme difference, the greater the influence of the process value on warpage and shrinkage, and the smaller the extreme difference, the smaller the influence of the process parameter on warpage and shrinkage, and the extreme difference equation is shown in Eq. (18).

$$ K_{mn} = \sum {Q_{mn} } $$

18 $$ k_{mn} = K_{mn} /4 $$

$$ R_{m} = k_{m\max } - k_{m\min } $$

where, $K_{mn}$——The sum of the values of the evaluation indicators at the level of the mth process parameter n, $Q_{mn}$——The value of the evaluation index at the level of the mth process parameter n, $k_{mn}$——The mean value of K_mn_, $R_{m}$——The nth process parameter extreme difference, $k_{m\max }$, $k_{m\min }$——The maximum and minimum values of the mean value at the mth process parameter.

#### Effect of each orthogonal experimental parameter of compression molding on warpage deformation

**Figure 11:**
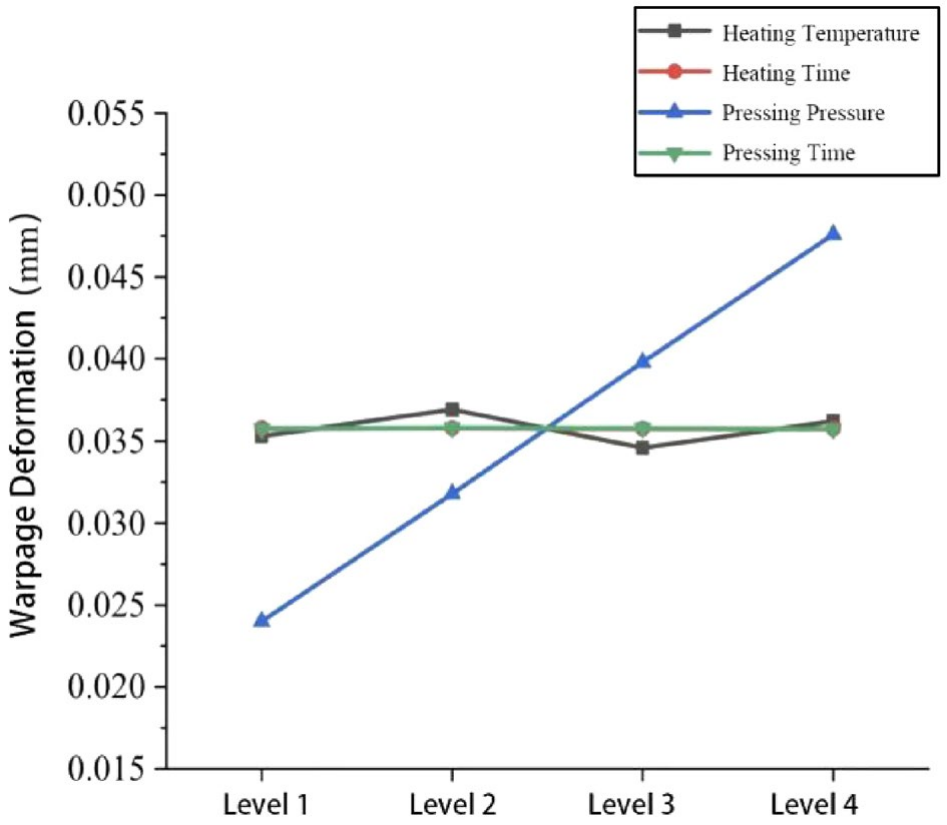
The degree of influence of each parameter on warpage deformation.

The mean value of each parameter on warpage deformation can be obtained from Eq. (18), as shown in Table 7. The degree of influence of each parameter on warpage deformation is shown in Fig. 11.

**Table 7:** Mean values of each parameter on warpage deformation.

Average value	Process parameters			
	A	B	C	D
*K *_1_	0.1413	0.1431	0.0958	0.143
*K *_2_	0.1477	0.1431	0.1272	0.1432
*K *_3_	0.1383	0.143	0.1589	0.1431
*K *_4_	0.1449	0.143	0.1903	0.1429
*K *_1_	0.0353	0.03578	0.0240	0.03575
*K *_2_	0.0369	0.03578	0.0318	0.0358
*K *_3_	0.0346	0.03575	0.0398	0.03578
*K *_4_	0.0362	0.03575	0.0476	0.03573
*R*	0.0023	0.00003	0.0236	0.00007
Sort	2	4	1	3

**Figure 12:**
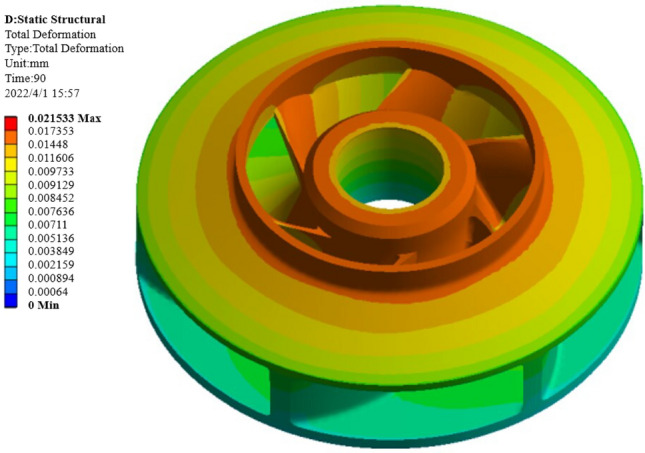
Analysis results under A3B3C1D4 process parameter combination.

From Table 4 and Fig. 11, it can be seen that the influence of the moulding process parameters on warpage deformation is pressing pressure (C) > heating temperature (A) > pressing time (D) > heating time (B), with the pressing pressure having the greatest influence on the warpage deformation of the closed plastic impeller. The best combination of moulding parameters is A3B3C1D4, i.e. heating temperature of 190 °C, heating time of 700 s, pressing pressure of 15 MPa and pressing time of 90 s. Simulation of the process parameters was carried out by ANSYS software to obtain the closed type plastic impeller with the minimum warpage, as shown in Fig. 12. The minimum warpage was 0.0215 mm, which corresponds to a wall thickness of 3.4 mm, and the data was substituted into Eq. (17) to obtain a volume shrinkage rate of 0.628%.

#### Effect of each compression molding process parameter on the shrinkage rate

**Figure 13:**
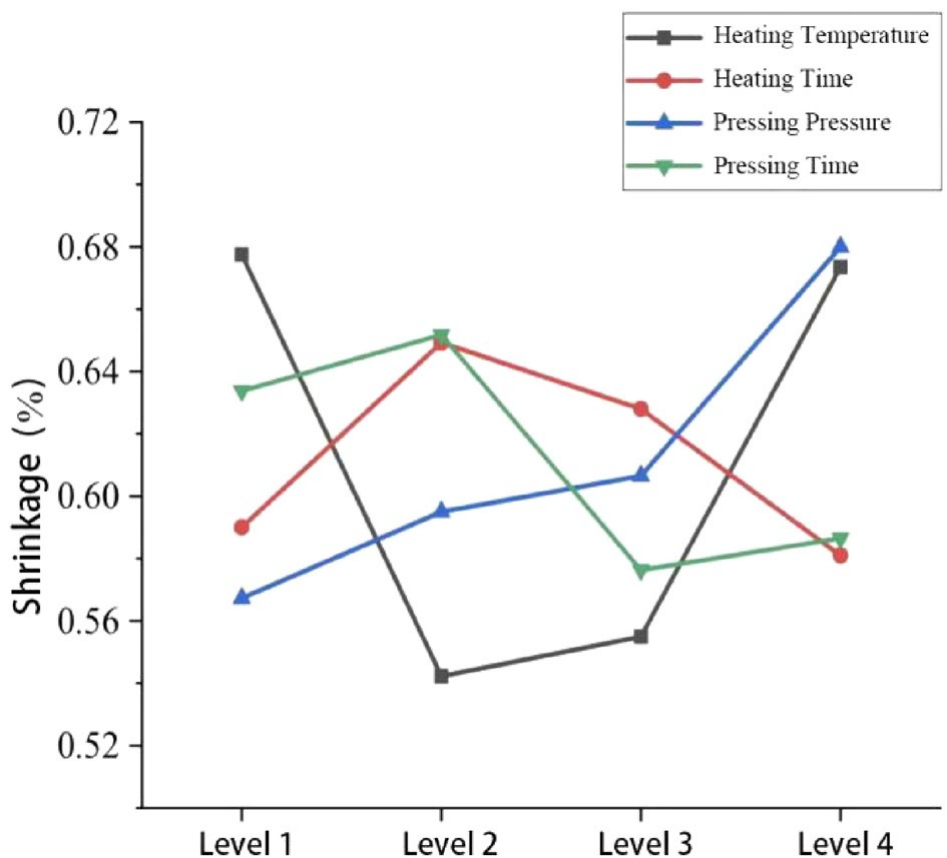
The degree of influence of each parameter on the shrinkage rate.

The mean value of each parameter on the shrinkage rate can be obtained from Eq. (18), as shown in Table 8. The degree of influence of each parameter on the shrinkage rate is shown in Fig. 13.

**Table 8:** Effect of each parameter on shrinkage rate.

Average value	Process parameters			
	A	B	C	D
*K *_1_	2.710	2.360	2.269	2.535
*K *_2_	2.169	2.597	2.378	2.607
*K *_3_	2.220	2.512	2.426	2.305
*K *_4_	2.694	2.324	2.720	2.346
*K *_1_	0.6775	0.5900	0.5673	0.6338
*K *_2_	0.5423	0.6493	0.5950	0.6518
*K *_3_	0.5550	0.6280	0.6065	0.5763
*K *_4_	0.6735	0.5810	0.6800	0.5865
*R*	0.1352	0.0683	0.1127	0.0755
Sort	1	4	2	3

**Figure 14:**
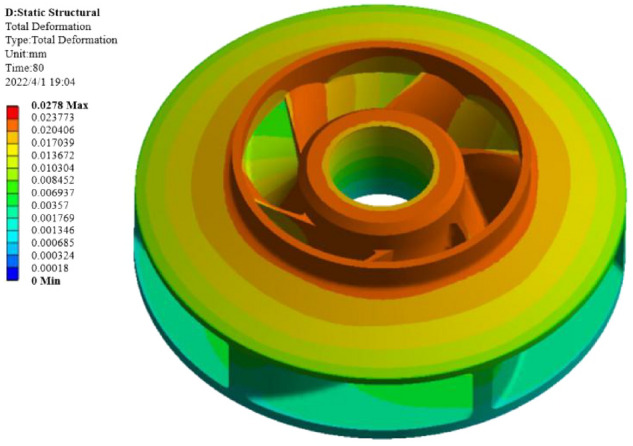
Analysis results under A2B4C1D3 process parameter combination.

Table 5 and Fig. 13 show that the influence of the moulding process parameters on the shrinkage rate is the heating temperature (A) > pressing pressure (C) > pressing time (D) > heating time (B), with the heating temperature having the greatest influence on the shrinkage rate of the closed type plastic impeller. The optimum combination of molding process parameters was A2B4C1D3, i.e., a heating temperature value of 180 °C, a heating time value of 800 s, a compression molding pressure value of 15 MPa, and a compression time value of 80 s. The process parameters were simulated by ANSYS software, and the warpage amount obtained is shown in Fig. 14. The warpage amount of 0.0278 mm and the corresponding wall thickness of 7 mm were substituted into Eq. (17) to obtain the minimum volume shrinkage of 0.398%.

## Optimization of process parameters based on GA-BP neural network and NSGA-II algorithm

The orthogonal experiment takes four levels for each of the four process parameters, and there is a large interval between each two levels. The sixteen data sets from the experiment cannot include all the values between the minimum and maximum levels of each process parameter. Therefore, the optimal combination of process parameters obtained from the orthogonal experiments is relatively rough, and the number between the maximum and minimum values of each level that has not been taken may be the best process parameter required. To obtain the optimal combination of process parameters, the GA-BP and NSGA-II algorithms were used to train the sixteen sets of orthogonal experimental data obtained^25^.

A GA-BP model for the prediction of the process parameters of closed plastic impeller compression molding was developed, which represents the corresponding relationships between all values between the minimum and maximum levels of the four process parameters and their resulting warpage deformation and shrinkage, and predicts the warpage deformation and shrinkage, respectively. The GA-BP model was verified to be correct by comparing the predicted values with the true values of the desired output ^26–28^. Then the data obtained from GA-BP were fitted using the global optimization-seeking function of the NSGA-II algorithm, which led to the optimal combination of process parameters for closed plastic impeller compression molding.

### Closed plastic impeller BP neural network structure design

Determine the number of BP neural network layers, the number of neurons in each layer, and the training function of each layer, to establish the structure of the closed plastic impeller BP neural network.

#### Determination of the number of network layers

The BP neural network model is to predict the results by varying the process parameters and is trained on the data obtained from orthogonal experiments of the closed plastic impeller. Therefore, a single implicit layer is sufficient to meet the current demand, and together with the input and output layers, a three-layer structure can be used in this paper.

#### Determination of the number of neurons in each layer

There are four closed plastic impeller compression molding process parameters and two evaluation indicators, so four neurons are selected for the input layer and two neurons for the output layer^29^.

The number of neurons in the hidden layer is obtained according to Eq. (19).

$$ m = \sqrt {n + t} + a $$

19 $$ m = \log_{2} n $$

$$ m = \sqrt {nt} $$

where, *m*—the number of design warp elements inside the implicit layer; *n*— the number of neurons in the input layer; *t*— the number of warp elements inside the output layer God; *a*— a constant from 1 to 10.

From *n* = 4 and *t* = 2, substitute into Eq. (19) and get *m* = 3 ~ 12.

The training error (e) is the difference between the ideal output y and the actual output u, as shown in Eq. (20).

20 $$ e = u - y $$

The mean squared error (E) is used in the BP neural network algorithm to represent the recognition accuracy, and it is concluded that the smaller the value of the mean squared error, the higher the accuracy, as shown in Eq. (21).

21 $$ E = \frac{1}{n}\sum\limits_{i = 1}^{n} {e_{i}^{2} } = \frac{1}{n}\sum\limits_{i = 1}^{n} {(u_{i} - y_{i} )^{2} } $$

**Figure 15:**
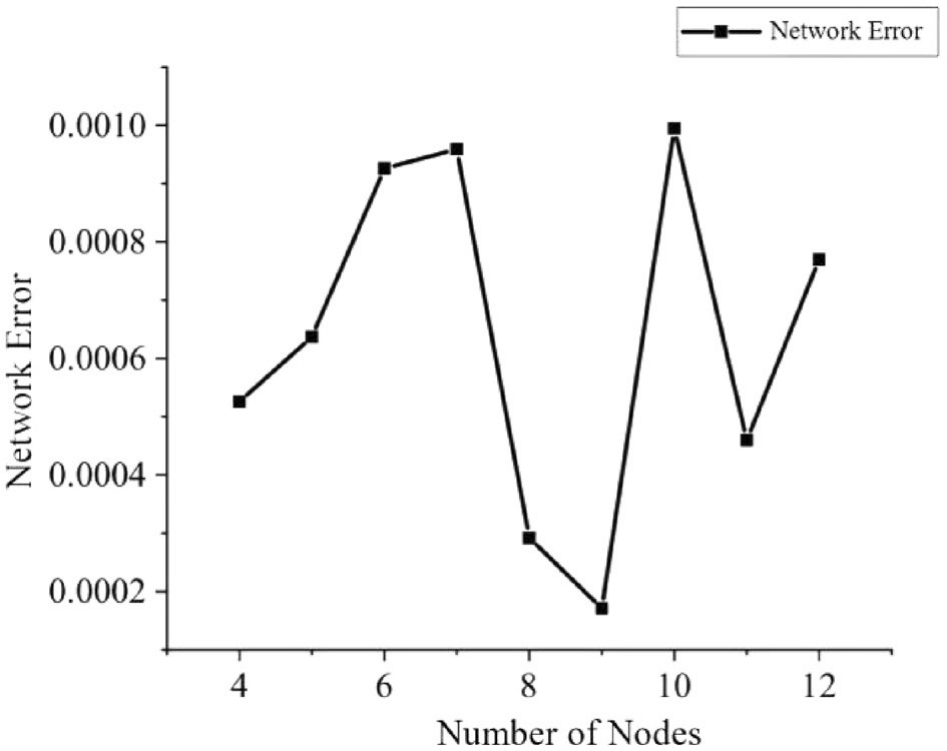
Error of different nodes within the hidden layer.

For GA-BP neural networks with different nodes inside the hidden layer, the results can be obtained by calculating their mean squared deviation; the higher the number of nodes, the smaller the network error in general, and the higher the overall accuracy, as shown in Fig. 15. When the number of nodes is 9, the network error reaches its minimum value, and the recognition accuracy reaches the highest position, so the final number of nodes in the hidden layer is chosen as 9.

#### Determination of the excitation functions for each layer

**Figure 16:**
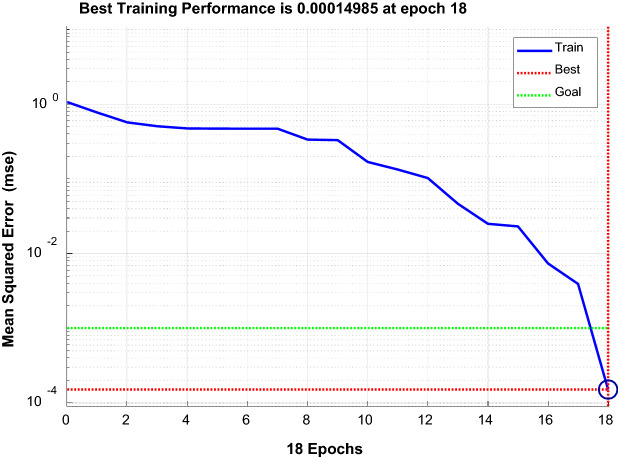
Trainlm performance curve.

**Figure 17:**
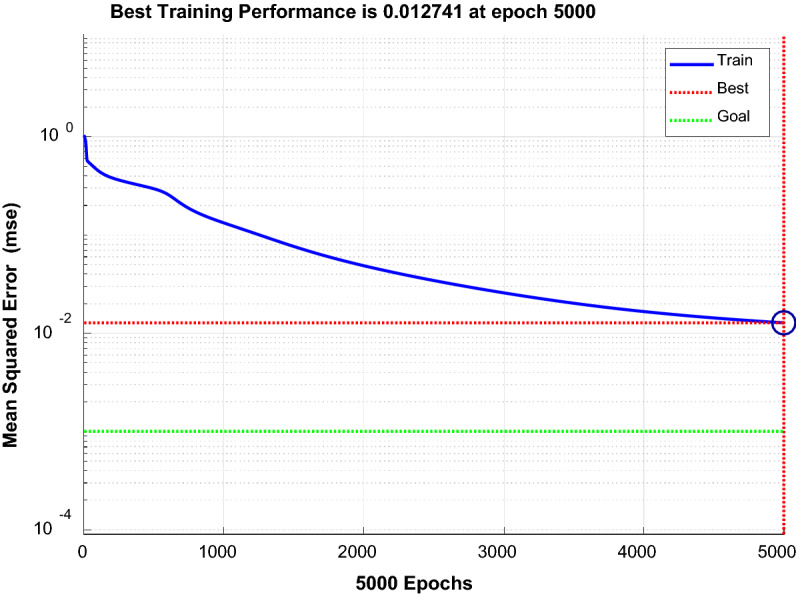
Traingd performance curve.

**Figure 18:**
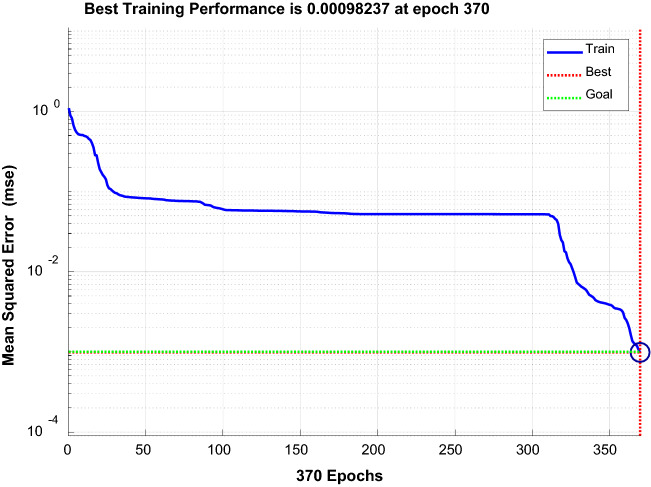
Trainbfg performance curve.

Tansig was selected for the implicit layer, and Purelin was selected for the output layer. The training functions were Traingd, Trainlm, and Trainbfg^30^. The performance curves of each function were compared, and the Trainlm function with fast convergence and small error was selected. Figure 16, Fig. 17, and Fig. 18 show the performance curves of the three functions, respectively.

**Figure 19:**
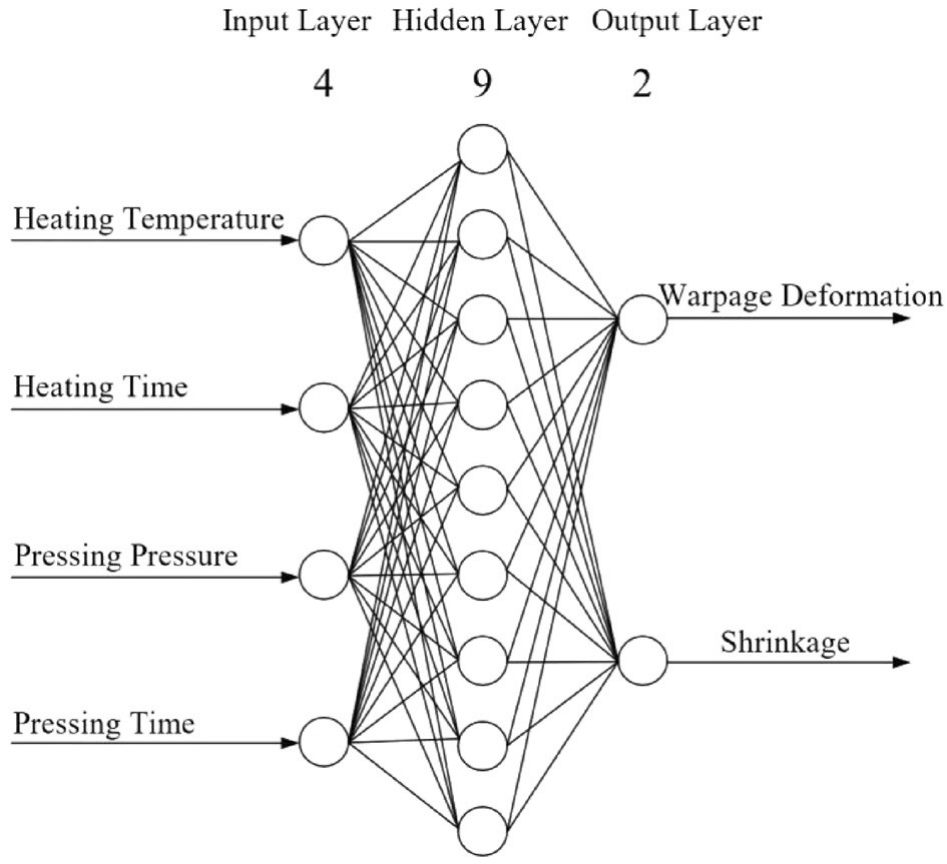
Neural network structure diagram.

The structure of the BP network created in this paper is shown in Fig. 19.

#### Data normalization process

The input data, definitions, and units are different, and to unify the samples, the data are normalized, as shown in Eq. (22).

22 $$ x^{\prime} = \frac{{x - x_{\min } }}{{x_{\max } - x_{\min } }} $$

Rewriting Eq. (22) allows for faster training, as in Eq. (23).

23 $$ x^{\prime} = 0.05 + 0.095\frac{{x - x_{\min } }}{{x_{\max } - x_{\min } }} $$

where, x_max_ ——maximum value; x_min_——minimum value.

The corresponding inverse normalization equation, as shown in Eq. (24).

24 $$ x^{\prime} = \frac{{(x^{\prime} - 0.05)(x_{\max } - x_{\min } )}}{0.95} + x_{\min } $$

### GA-BP neural network structure

Using the genetic algorithm to optimize the weights and thresholds of the BP neural network can prevent the BP neural network from falling into local optimum and can significantly accelerate its convergence speed. Choosing the appropriate population size can make the computation speed not too slow, and the computation error can be more reasonable. In general, the population size is between 10 and 200, and the population size in this paper is 50, according to the actual choice.

The selection operation uses roulette to simulate the survival of the fittest, and the better individuals are retained as much as possible. Variation and crossover are effective methods to avoid falling into local optimum, in which crossover is mainly used to improve population diversity and variation is used as a supplement. The crossover rate is chosen to be 0.7, and the variation rate is chosen to be 0.1, which can achieve a faster convergence speed and ensure global superiority seeking.

### GA-BP neural network sample training and results

**Figure 20:**
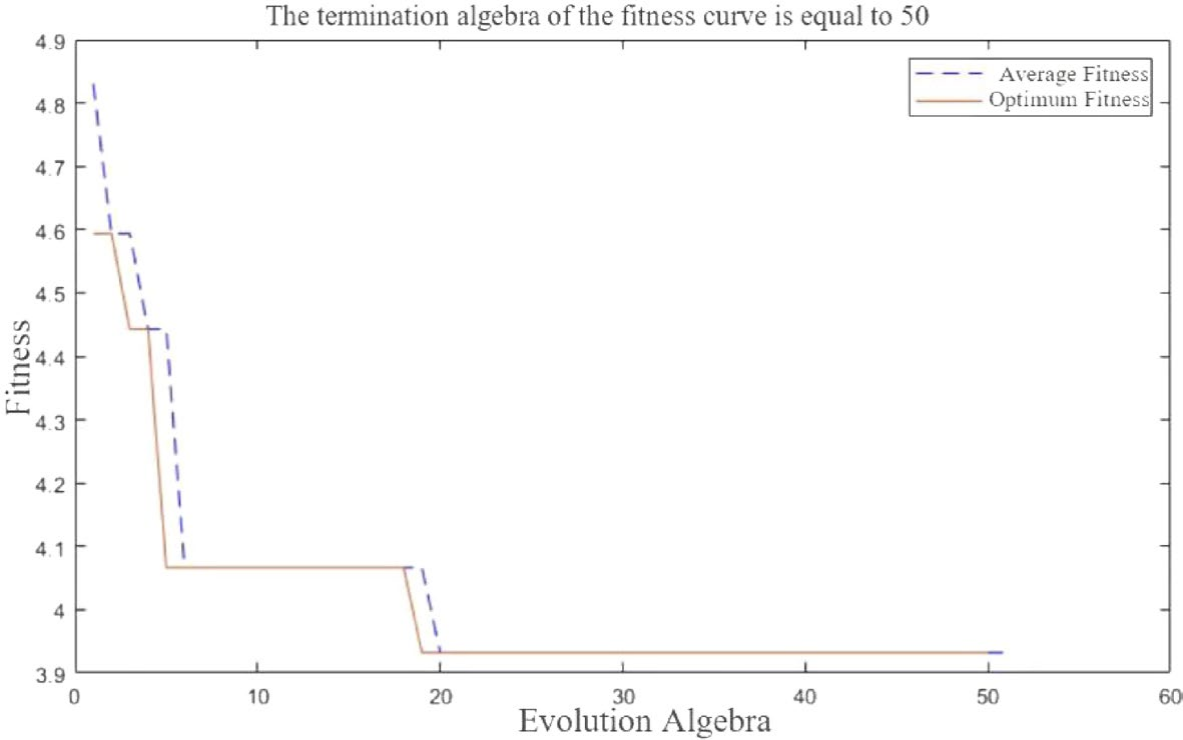
Convergence graph of training iterations.

The larger the proportion of training samples, the better the results. Generally, 4/5 of the sample data is used for training. There are 16 groups of data in the experiment. 12 groups of data are randomly selected as training samples, which are 2, 3, 4, 6, 7, 9, 10, 11, 13, 14, 15 and 16, respectively. The remaining four data groups are test samples, which are 1, 5, 8 and 12, respectively. The iteration algebra is 50 generations, and convergence is achieved after 19 iterations, as shown in Fig. 20.

**Figure 21:**
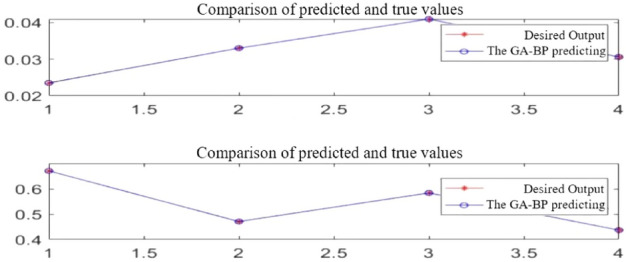
Comparison of GA-BP predicted and true values.

The comparison of the predicted and true values of warpage deformation and shrinkage obtained for the four process parameters is shown in Fig. 21. As can be seen from the figure, the curves of the GA-BP predicted values and the true values basically overlap, indicating that the GA-BP neural network has a high accuracy to meet the demand.

### GA-BP neural network predicts the influence of process parameters on evaluation indexes

**Figure 22:**
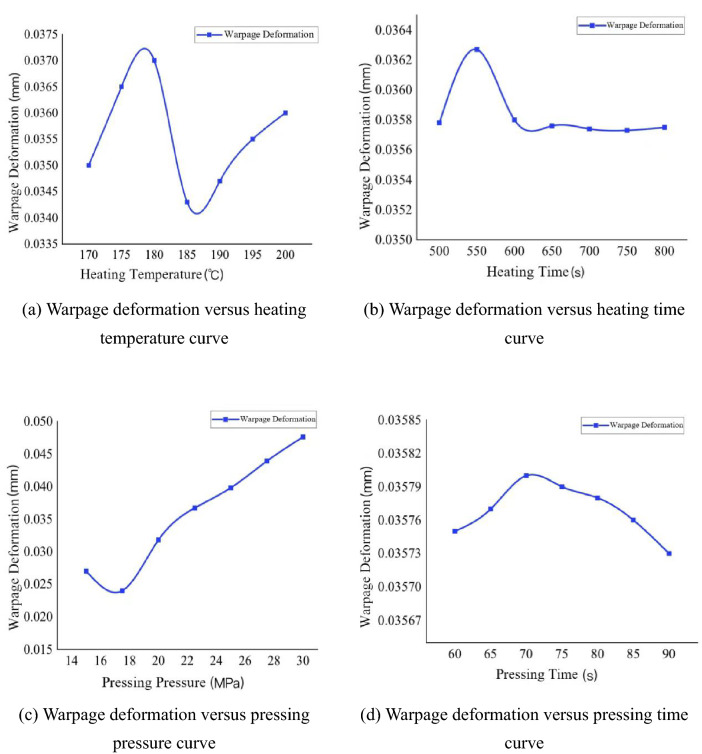
Warpage deformation curve with process parameters.

**Figure 23:**
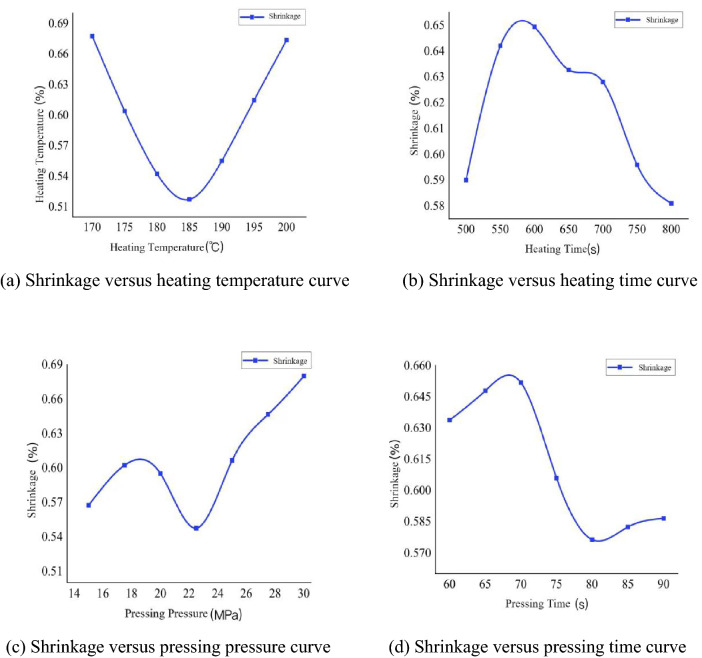
Variation curve of shrinkage rate with process parameters.

To study the trend of impeller evaluation index changing with process parameters, the minimum volume shrinkage and warpage under the condition of changing a single process parameter are predicted by using the prediction function of the established neural network based on the optimal process parameter combination, and the effects of heating temperature, heating time, pressing pressure and pressing time on the warpage deformation and shrinkage are obtained, as shown in Fig. 22 and Fig. 23.

As can be seen from Fig. 22, the influence of the critical parameters of the forming process on the warpage deformation through neural network analysis is as follows: as the heating temperature increases, the warpage deformation first becomes larger, then smaller, and then larger, with the optimal value between 180 °C and 190 °C; as the heating time increases, the warpage deformation first becomes larger, then smaller and then stable, with the optimal value after 600 s; as the pressing pressure increases, the warpage deformation first becomes smaller and then larger, with the optimal value between 15 and 20 Mpa; as the pressing time increases, the warpage deformation first becomes larger and then smaller, but the pressing time is not too long, with the optimal value between 15 and 20 Mpa. With the increase of pressing pressure, the warpage deformation first becomes smaller and then larger; the optimal value is between 15 and 20 Mpa; with the increase of pressing time, the warpage deformation first becomes larger and then continues to become smaller, but the pressing time is not too long, the optimal value is around 90 s.

As can be seen from Fig. 23, the neural network analysis of the critical parameters of the molding process on the impact of shrinkage rate, it is concluded that: with the increase of heating temperature, shrinkage first becomes smaller and then becomes larger; the optimal value between 180 °C and 190 °C; with the increase of heating time, shrinkage first becomes larger and then becomes smaller, but the heating time is not too long, the optimal value between 750 and 800 s; with the increase of pressing forming pressure, shrinkage first becomes larger and then becomes smaller and larger, the optimal value between 20 and 25 Mpa; with the increase of pressing time, warpage deformation first becomes larger and then becomes smaller and stabilizes, the optimal value between 80 and 90 s.

### NSGA-II algorithm-based process parameter set solving

**Figure 24:**
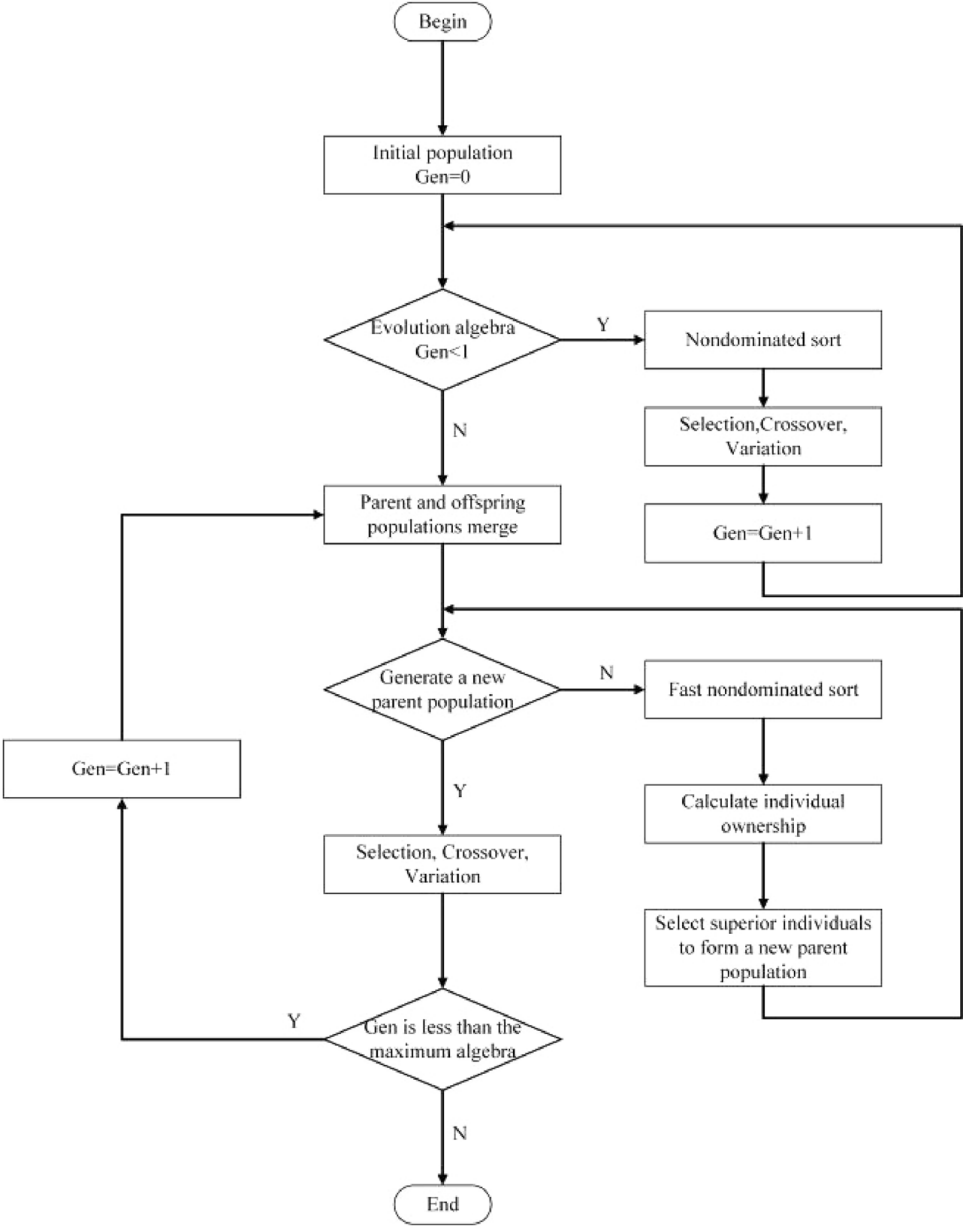
NSGA-II algorithm flow chart.

The NSGA-II algorithm has good convergence in dealing with multi-objective optimization aspects of the problem^31,32^. In this paper, the NSGA-II algorithm is applied to derive the optimal combination of process parameters for closed plastic impeller compression molding with warpage deformation and shrinkage as the objectives. The NSGA-II algorithm in dealing with the multi-objective optimization process^33^, as shown in Fig. 24.

#### Data fitting and multi-objective optimization

**Figure 25:**
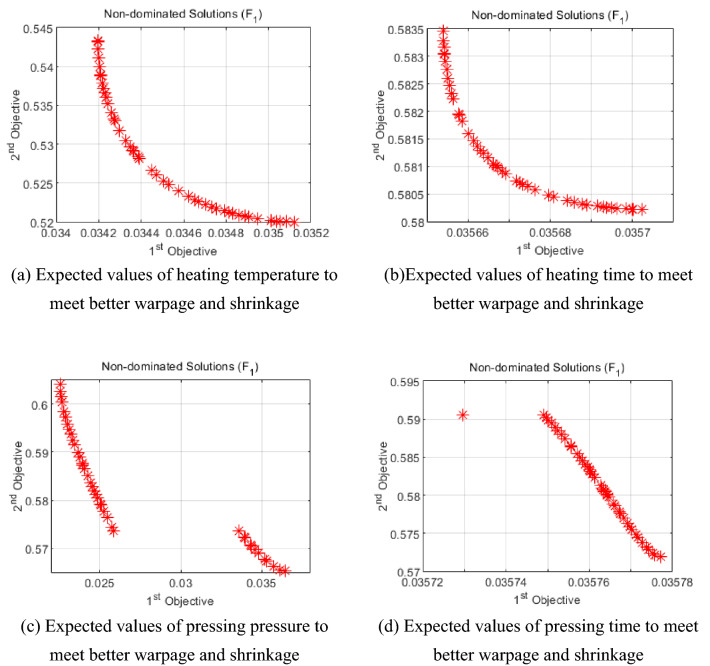
Expected values for a certain process parameter with good warpage and shrinkage.

The data predicted by the GA-BP neural network were functionally fitted by the polyfit function in MATLAB, and the NSGA-II algorithm was used for multi-objective optimization^34^. With a population size of 50, 200 iterations, a crossover probability of 0.85, and a variance probability of 0.1, the expectation values under a certain process parameter were solved separately to obtain better warpage deformation and shrinkage, as shown in Fig. 25.

#### Selection of weights and output of results

**Figure 26:**
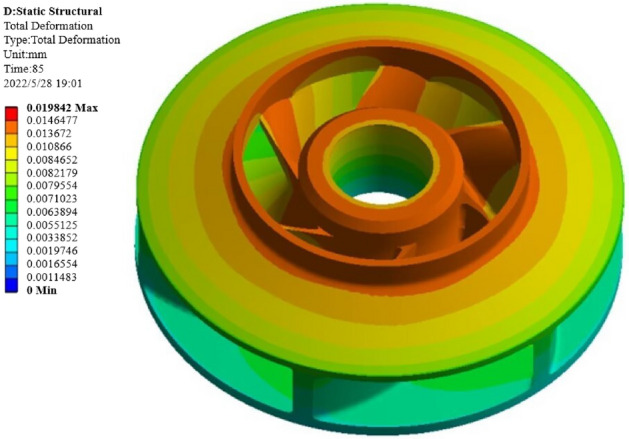
Warpage amount after optimization by NSGA-II algorithm.

The warping deformation and shrinkage are two evaluation indexes that are very important for the molding quality of the closed plastic impeller, so the weight of warping deformation and shrinkage is set to 0.5. After the NSGA-II algorithm is used to solve the problem, the output feasible solution set results are subjected to data processing and screening, and the best combination of process parameters is finally determined as [187, 785, 19, 85], that is, the heating temperature is 187 °C, the heating time is 785 s, the pressing pressure is 19 Mpa, and the pressing time is 85 s.By combining the molding process parameters for simulation, the warpage of the impeller is 0.0198, as shown in Fig. 26. The warpage is 0.0198 mm, and the wall thickness of the spot workpiece is 5.6 mm. The data is substituted into Equation (17) to obtain a volume shrinkage of 0.353%.

The warpage and shrinkage before and after optimization by the NSGA-II algorithm are compared, as shown in Table 9.

**Table 9:** Comparison of optimization results of NSGA-II algorithm.

Optimization method	Process parameters			Optimization results		
	Heating temperature (°C)	Heating time (s)	Pressing pressure (MPa)	Pressing time(s)	Warpage deformation (mm)	Shrinkage (%)
Warpage optimization based on orthogonal test	190	700	15	90	0.0215	0.628
Shrinkage optimization based on orthogonal test	180	800	15	80	0.0278	0.398
Optimization results based on NSGA-II algorithm	187	785	19	85	0.0198	0.353

It can be seen from Table 9 that the warpage of the impeller optimized by the NSGA-II algorithm is reduced from 0.0215 mm to 0.0198 mm, and the shrinkage is reduced from 0.398 to 0.353%. It shows that the NSGA-II algorithm can get better results by optimizing process parameters, and can get a group of process parameters with optimal warpage and shrinkage at the same time.

## Conclusion

1. Based on the theoretical equation of pressing pressure for thin sheet-type plastics, the theoretical derivation of pressing pressure suitable for cylindrical cavities was obtained, and the theoretical derivation of pressing pressure suitable for closed plastic impeller molding was obtained. By calculation, it is obtained that the pressing pressure of closed type plastic impeller is 15 MPa.
2. The heating temperature, heating time, compression molding pressure, and compression time were determined as a set of process parameters affecting the molding of the closed plastic impeller, and the warpage deformation and shrinkage rate were used as indicators to evaluate the goodness of the product. The orthogonal experiment table was designed, and the orthogonal test results of closed type plastic impeller compression molding were obtained by numerical simulation of the heating process and compression molding process of plastic raw materials through ANSYS.
3. The orthogonal test results of closed type plastic impeller compression molding were analyzed to obtain the influence trend of each process parameter on warpage deformation: compression molding pressure (C) > heating temperature (A) > pressing time (D) > heating time (B) and the optimal combination of warpage deformation compression molding process parameters A3B3C1D4. The influence trend of each process parameter on shrinkage rate: heating temperature (A) > pressing pressure (C) > pressing time (D) > heating time (B) and the combination of optimal compression molding process parameters for shrinkage A2B4C1D3.
4. The prediction model of the GA-BP neural network for closed plastic impeller was determined, and a set of process parameter combinations satisfying both warpage deformation and shrinkage were obtained by the multi-objective search function of the NSGA-II algorithm: heating temperature of 187 °C, the heating time of 785 s, pressing pressure of 19 MPa, and pressing time of 85 s.

## Data Availability

The data used to support the findings of this study are included within the article.
